# Body Weight Changes During Ramadan Intermittent Fasting: A Cross-Sectional Study of Healthy Adults in Turkey

**DOI:** 10.1155/jnme/8851660

**Published:** 2025-02-05

**Authors:** Ammar Mektebi, Mağfiret Abdulveli Bozlar, Noura Kanjo, Muhammed Munir Al-Jebaili, Youssef Nasrallah, MoezAlIslam Faris, Moien AB Khan, ISRI Research Group

**Affiliations:** ^1^Faculty of Medicine, Kutahya Health Sciences University, Kutahya, Türkiye; ^2^Department of Public Health, Hamidiye International School of Medicine, University of Health Sciences, Istanbul, Türkiye; ^3^Faculty of Medicine, Ondokuz Mayis University, Samsun, Türkiye; ^4^Hamidiye International Faculty of Medicine, University of Health Sciences, Istanbul, Türkiye; ^5^Faculty of Medicine, Kahramanmaraş Sütçü İmam University, Kahramanmaraş, Türkiye; ^6^Department of Clinical Nutrition and Dietetics, Faculty of Allied Medical Sciences, Applied Sciences Private University, Amman, Jordan; ^7^Health and Wellness Research Group, Department of Family Medicine, College of Medicine and Health Sciences, United Arab Emirates University, Al-Ain, UAE

**Keywords:** calorie restriction, intermittent fasting, lifestyle, time-restricted eating, Turkey, weight changes

## Abstract

**Introduction:** This study explores the impact of observing Ramadan intermittent fasting (RIF) on body weight in Turkish residents, marking it as the first study conducted in Turkey to investigate the interplay of religious fasting with dietary changes amid the COVID-19 pandemic. We hypothesized that observing Ramadan fasting would result in weight loss attributable to dietary changes, while decreased physical activity would correlate with weight gain during this period.

**Methods:** A cross-sectional survey was conducted among Muslims aged 18+ in Turkey who fasted at least two days during Ramadan 2021. Data were collected via Google Forms, with 1669 participants recruited through social media. Descriptive statistics summarized participant characteristics, and Pearson's Chi-square tests assessed weight change differences. Binary logistic regression identified predictors of weight gain, adjusting for factors such as sex, age, physical activity, water consumption, and diet.

**Results:** Of the 1669 respondents (53.6% female), 49.4% perceived their weight as normal, while 47.0% classified themselves as overweight or obese. Logistic regression indicated that decreased physical activity (AOR = 1.618, *p*=0.001) and increased fat intake (AOR = 1.9, 95% CI 1.2–2.9) were significant predictors of weight gain.

**Conclusion:** Our findings emphasize the importance of promoting healthy eating and regular physical activity during Ramadan.

## 1. Introduction

Obesity is a major risk factor for many noncommunicable diseases (NCDs), which have a significant economic and societal impact and can lead to major comorbidities and mortality [1]. The current evidence suggests that dietary and lifestyle modifications such as fasting, with emphasis on intermittent fasting (IF), caloric restriction, weight-reducing diets, and regular physical activity can reverse or protect against the adverse metabolic disruptions linked to obesity [2, 3].

Ramadan is the ninth month of the Islamic lunar calendar, during which Muslims are mandated to refrain from consuming food and drink from dawn till sunset for 29–30 consecutive days. Being a lunar month, Ramadan fasting may occur during any of the solar months, leading to a wide range of fasting hours extending from 12 up to 21 h depending on the geography and solar season, allowing 4–12 eating hours. This is typically like the time-restricted eating (TRE) regimen [4]. Ramadan IF (RIF) has recently attracted attention for its potential effects on body physiology and metabolism [5–8]. Among these, RIF is associated with significant changes in total dietary intake of energy and macronutrients, accompanied by major changes in food group consumption, and associated with significant improvements in body composition and weight, particularly in body fatness and visceral fat, with improved metabolic syndrome components, liver function tests, glucometabolic markers, as well as inflammatory and oxidative stress markers. Muslims typically eat one large meal after sundown and a smaller meal before dawn during Ramadan, although some may also eat another meal before bedtime [3, 9–23].

Physiological changes observed during RIF, are likely attributed to alterations in eating, and sleeping habits and changes in physical activity patterns [24–26]. Muslims tend to eat a wider variety of food during Ramadan than they do throughout the year [24, 27]. Consequently, dietary patterns and meal timing may significantly change during this fasting period, affecting weight and other health consequences.

It is crucial to understand how RIF affects body weight and associated dietary changes to promote health and provide appropriate guidance to those who observe this religious practice. Dietary patterns and meal timing undergo significant changes during RIF, potentially affecting weight and overall health. In several systematic reviews and meta-analyses, RIF has been assessed for its impact on body weight, but findings have been mixed.

In a comprehensive systematic review and meta-analysis of 85 studies, including 4176 healthy subjects (aged 16–80 years) and conducted in 25 countries from 1982 to 2019, the observance of RIF yielded a slight significant reduction in body weight equivalent to −1.022 kg. The high heterogeneity in the obtained results was directly attributed to the factors associated with the observance of fasting, such as fasting time, food consumption patterns and daily activities, the season, and geographical location (country) [3]. Similarly, another meta-analysis, despite the variations in fasting-related factors, found a significant reduction in body weight and body fat percentage among healthy nonathlete adults [14].

In a large-scale, multicountry cross-sectional study across 27 countries during the COVID-19 pandemic and using structural equation modeling over 24,541 enrolled adults, it was found that many factors interplayed in determining the changes in sleep quality and quantity during Ramadan. Among these, physical activity patterns, consumption of plant-based proteins, smoking, consumption of vegetables, fruits, and plant-based proteins, consumption of ready-to-eat delivered food, and infrequent screen time were all variably associated with different sleep quality levels among the study participants [28].

Amid the COVID-19 pandemic, the Turkish government implemented a series of restrictions on individual movements during Ramadan 2021, including banning the organizing of large gatherings, such as traditionally accustomed prefasting meals *Suhoor* events or the sunset, fasting-breaking meals *Iftar* tents, to restrict the contingency of the virus. Moreover, restaurants and cafes were limited to providing takeaway services only, affecting the dining habits of fasting individuals [29]. The changes in lifestyle and eating habits during Ramadan combined with the additional confinement measures due to the pandemic, raised concerns about people's overall well-being during Ramadan 2021. However, Ramadan is also seen as an opportunity for Muslims to make meaningful lifestyle modifications, promote self-discipline and self-control, and foster happiness and better health for themselves and their loved ones. Despite these benefits, the unique circumstances of Ramadan 2021 created a general apprehension about the lifestyle and dietary choices of individuals.

As a result of the COVID-19 pandemic, researching the impact of RIF on dietary changes has been challenging and limited. The global health crisis has significantly affected Ramadan, including the associated lockdown measures and social distancing protocols. As a result of these circumstances, there are notable gaps in the literature regarding how RIF affects dietary patterns and nutritional intake in such cases. In addition, the related studies [30, 31], however, were limited to specific regions or had small sample sizes, which hindered their generalizability. For this reason, it is imperative to conduct a comprehensive nationwide assessment of RIF-related dietary changes and their association with body weight among healthy residents in Turkey. Our study addresses this gap by examining the changes in body weight during the fasting month and exploring the underlying dietary and some lifestyle modifications that occurred among Turkish residents during the COVID-19 pandemic. Specifically, we aimed to determine how Ramadan fasting can affect weight gain, emphasizing the importance of mindful eating habits and the consumption of healthier food choices.

## 2. Methods

### 2.1. Study Design, Setting, and Participants

As part of a broader international study, an online cross-sectional survey was conducted from May 9, 2021 (the 27th day of Ramadan) to June 4, 2021, during this period, Ramadan fasting hours in Turkey varied between 15 and 16 h. The target population included Muslims aged 18 or older who were living in Turkey and had fasted or at least two days during Ramadan. Individuals following specific dietary regimens or working night shifts were excluded from the study, as specified in the instructions at the beginning of the electronic questionnaire.

The survey was administered using Google Forms and distributed by data collectors across eight different cities in Turkey: Istanbul, Samsun, Kütahya, Mersin, Kayseri, Gaziantep, Bursa, and Antalya. To maximize participation, the survey link was shared on social media platforms like WhatsApp, Facebook, Twitter, and Instagram, allowing the invitation to reach a wide audience beyond the initial cities.

### 2.2. Ethical Considerations

This research was carried out following the principles outlined in the Declaration of Helsinki [32] and was approved by the research ethics committee (REC) of the Istanbul Health Sciences University, Istanbul, Turkey (E-46418926-050.01.04-75042), and the Social Sciences REC (REC) of the United Arab Emirates University (Approval Number ERS_2021_7308). All participants provided informed consent by accepting the terms on the Google Forms platform prior to participation. Their involvement was entirely voluntary, with no monetary or nonmonetary incentives provided.

### 2.3. Study Instrument and Data Collection

The data were collected anonymously, without any identifying information. A web-based, self-administered electronic questionnaire that was pretested and adapted from previously validated questionnaires [28, 31, 33] was used for data collection. The translation and cultural adaptation process followed the “Principles of Good Practice for the Translation and Cultural Adaptation Process” [34]. Initially, forward translations were performed by two independent translators fluent in both English and Turkish, the primary language of the study. These translations were then back-translated into English for review. After reviewing the back-translations, the Turkish version of the questionnaire was proofread and edited as necessary. It was then pilot-tested with 30 participants to ensure clarity and appropriateness. Following this, the final version of the Turkish questionnaire was used. Arabic and English translations were added to increase accessibility for participants, but the primary language of the study remained Turkish, reflecting the majority language of Turkey. The reliability of the questionnaire was determined by calculating Cronbach's alpha for scales measuring dietary modification, weight change, and perceived health status. In this study, all scales were reliable, with a Cronbach's alpha of 0.80. Respondents were provided with a unique Google Form link that could be disseminated via social media. To include all Muslims living in Turkey, English and Arabic translations of the questionnaire were added to the questionnaire according to Helsinki Guidelines [32]. All survey responses were automatically collected into a single Google spreadsheet for streamlined data cleaning and analysis.

### 2.4. Sample Size Calculation

The sample size was calculated using Epi Info software (version 7.2.4.0), based on the estimated Turkish Muslim population of approximately 81.7 million in 2021 [35]. We applied the following parameters to ensure statistical robustness: a 5% margin of error, a 99.9% confidence level, and a significance level of 0.5. Using these parameters, the minimum required sample size was determined to be 1514 participants. All individuals meeting the inclusion criteria were invited to participate in the study, resulting in data collection from 1669 participants. This number exceeded the original target, further enhancing the representativeness of the sample and the robustness of the study's findings.

### 2.5. Description of Variables

The survey questionnaire collected data on participants' sociodemographic characteristics, dietary habits, dietary diversity, weight fluctuations, water consumption, and physical activity both before and during Ramadan fasting.

#### 2.5.1. Sociodemographic Variables

These included sex, age, nationality, region of residence, marital status, living area, and living conditions. The number of fasting days experienced during Ramadan was also recorded. These sociodemographic factors served as explanatory variables, hypothesized to influence dietary habits and fasting behaviors.

#### 2.5.2. Dietary Habits and Eating Behaviors

Dietary habits and eating behaviors were assessed through questionnaires focusing on changes since the onset of fasting during Ramadan. Participants reported alterations in the quality and quantity of food consumed, as well as modifications in dietary diversity, including the intake of vegetables, fruits, grains, dairy, and meat. Daily water consumption was also evaluated, distinguishing between sufficient (more than 3 cups) and insufficient (3 cups or less) intake. Snacking habits between *Iftar* and *Suhoor*, as well as changes in physical activity (whether increased, decreased or unchanged), were also recorded. These variables were treated as explanatory factors to analyze their impact on overall health and fasting-related behaviors during Ramadan.

#### 2.5.3. Outcome Variables

The outcome variables focused on participants' self-reported weight and height, which were used to calculate body mass index (BMI), categorized as underweight (< 18.5 kg/m^2^), normal weight (18.5–24.9 kg/m^2^), overweight (25.0–29.9 kg/m^2^), or obese (≥ 30 kg/m^2^). Participants also indicated perceived weight changes during Ramadan, with options for weight loss (ranging from −1 to −4.5 kg or more), maintenance (0 kg), or weight gain (ranging from +1 to +4.5 kg or more). Water consumption, alongside dietary habits and physical activity, was analyzed to understand its influence on weight fluctuations and overall health during Ramadan.

### 2.6. Data Analyses

Data analyses were conducted using SPSS version 21 (IBM SPSS Statistics for Windows, Version 21.0, Armonk, NY: IBM Corp; 2012). The demographic and behavioral characteristics of the participants were summarized through descriptive statistics. Age was categorized into predefined groups, including 18–24, 25–34, 35–44, 45–54, 55–64, and 65–74 years. Categorical variables—including sex (female and male), marital status (divorced, married, single, and widowed), area of residence (city, town, or village), and perceived weight status (extreme overweight or obese and normal weight)—were summarized using frequencies and percentages. Frequencies and percentages were also calculated for food group consumption during Ramadan. To assess differences in weight change, Pearson's Chi-square test was used. Proportions of participants reporting no weight change, weight loss, and weight gain were calculated, with 95% confidence intervals (CIs) provided. The analysis included only participants who reported their pre-Ramadan weight and those who fasted for more than 20 days during the month. To further investigate predictors of weight gain, binary logistic regression was utilized, adjusting for potential confounding variables including sex, age, water consumption, and physical activity. Dietary predictors of weight gain were also analyzed through binary logistic regression, with a significance level set at *p* < 0.05. Odds ratios (ORs) and 95% CIs were employed to interpret the results of the regression models.

## 3. Results

### 3.1. Participant Characteristics

As presented in Table 1, a total of 1669 people responded to the questionnaire; more than half (53.6%) of them were women. The age range with the highest representation in this sample was 18–24. Concerning perceived weight, 49.4% of respondents classified their weight as normal weight and 47.0% as overweight or obese. The majority of them (83.2%) fasted for a duration ranging from 21 to 30 days.

### 3.2. Change in Consumption of Different Types of Food During Ramadan

**Figure 1:**
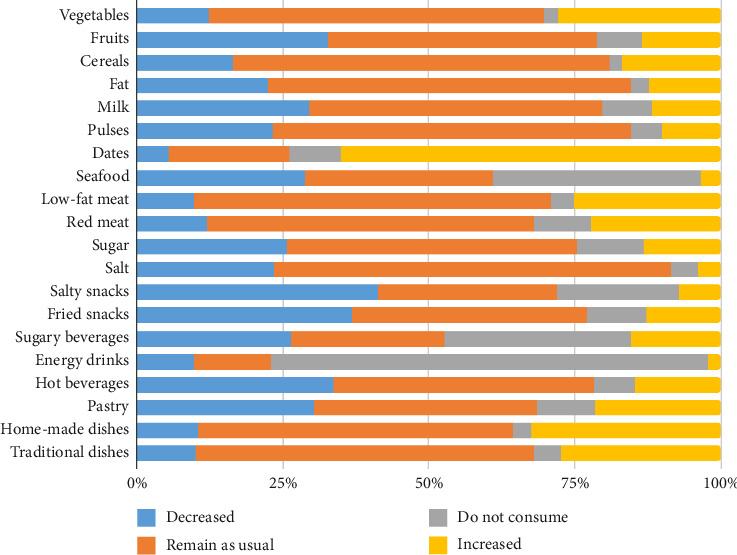
Modification of food groups during Ramadan.

Figure 1 shows that regarding food consumption during Ramadan, the increased food consumption was mostly observed for palm dates (66% *n* = 1085), while others reported increases in consumption of homemade dishes (32% *n* = 542) and vegetables (28% *n* = 463). The consumption of salty snacks (41% *n* = 690), fried snacks (37% *n* = 614), fruits (33% *n* = 546), milk products (30% *n* = 495), and seafood (29% *n* = 481) decreased. A high proportion of the respondents maintained their amount of consumption of salt (68% *n* = 1132), cereals (65% *n* = 1077), fat (62% *n* = 1037), pulses (61% *n* = 1023), low-fat meat (61% *n* = 1021), traditional dishes (58% *n* = 969), and sugar (50% *n* = 826).

### 3.3. Weight Change Patterns and Associated Factors During Ramadan

Table 2 presents the weight change patterns among participants, showing that 44.2% experienced weight loss, 37% reported no weight change, and 18.8% reported weight gain. The analysis revealed significant associations with various factors. While females showed a higher proportion of weight loss (45.9%) compared to males (42.2%), this difference was not statistically significant (*p*=0.124). Age was significantly associated with weight change (*p*=0.005), with participants aged 35-44 demonstrating the highest weight loss (60.0%). Physical activity also significantly influenced weight change (*p* < 0.001); participants with decreased physical activity had the highest weight gain (21.1%), while those with increased physical activity reported a higher weight loss (52.3%). Snacking frequency between *Iftar* and *Suhoor* significantly impacted weight changes (*p* < 0.001), with infrequent snackers experiencing the most weight loss (48.6%). However, water consumption did not show a significant association with weight change (*p*=0.272).

### 3.4. Binary Logistic Regression Analysis of Weight Gain During Ramadan

Table 3 presents the results of the binary logistic regression analysis assessing the likelihood of weight gain during Ramadan, adjusted for sex, age, water consumption, and physical activity. Participants who reported decreased physical activity had a higher likelihood of weight gain (AOR = 1.618, *p*=0.001). In contrast, those who increased their physical activity did not show a significant association with weight gain (AOR = 1.166, *p*=0.525). The analysis indicated that sex and age did not significantly predict weight gain; specifically, females had an OR of 1.155 (*p*=0.312), and the age group of 35–44 years showed an OR of 0.813 (*p*=0.561) compared to the reference group of 18–24 years. Notably, the 65–74 age group exhibited significantly lower odds of weight gain (AOR = 0.142, *p* < 0.001). Water consumption also did not demonstrate a significant association with weight gain (AOR = 0.840, *p*=0.281).

### 3.5. Dietary Choices as Predictors of Weight Gain During Ramadan

Table 4 shows that fat and sugary beverage consumption were significant predictors of weight gain during Ramadan. Respondents who consumed more fat during this month were almost two times more likely to gain weight than those who did not change their intake or ate less fat (AOR: 1.9, 95% CI: 1.2–2.9). Furthermore, the odds of gaining weight during Ramadan among respondents who ate more sugary beverages were higher than those who did not change their intake or ate less sugary beverages (AOR: 1.7, 95% CI: 1.2–2.5).

## 4. Discussion

This study aimed to investigate the effects of RIF on body weight among healthy adults in Turkey, particularly during the unique context of the COVID-19 pandemic, and to explore any factors that may lead to unexpected weight gain during the month. Our findings indicate that several factors significantly influence weight change during Ramadan. Specifically, participants who were less physically active and those who consumed higher amounts of fats and sugary beverages were more likely to experience weight gain during this month. Thus, these factors highlight the complexity of how fasting during Ramadan can impact body weight depending on individual habits.

Previous research has reported mixed results regarding the impact of Ramadan fasting on body weight. A systematic review and meta-analysis reported a reduction in body weight (−1.34 (95% CI: −1.61 to −1.07) kg, *p* < 0.001) and body fat (−1.46 (95% CI: −2.57 to −0.35)%, *p*=0.010) among individuals with overweight or obesity [14]. According to the authors, body weight reduction was probably attributed to an increase in energy expenditure [14]. However, another study showed a limited impact of Ramadan fasting on body weight and fat with a decrease of less than 5% in both parameters in people with normal weight. This reduction was attributed to lower energy intake during this period [36, 37]. Our study adds nuance to these findings by highlighting that dietary habits, particularly the consumption of high-calorie foods and sugary beverages, can mitigate the potential weight-reduction benefits of fasting, leading to weight gain instead.

These results agree with the evidence that adopting dietary patterns that prioritize vegetables, fruits, whole grains, seafood, legumes, moderate amounts of dairy products (particularly low and nonfat options), and alcohol, while limiting the intake of meats (including red and processed meats), sugar-sweetened foods and beverages, and refined grains, can have favorable outcomes in terms of body weight, such as lower BMI, waist circumference, or percent body fat, and reduced risk of obesity [38, 39]. The findings emphasize the importance of individuals being mindful of their dietary choices and aiming for a balanced and healthy diet during Ramadan to minimize the risk of weight gain.

Moreover, while previous research has highlighted sex disparities in weight loss during RIF, with men often experiencing greater weight loss than women [33, 40], our data did not reveal any significant differences between genders in weight changes, suggesting that further investigation is needed in this area.

One concerning finding from our study is that over one-third of the respondents, particularly within the younger age group of 21–30 years, are either overweight or obese. This aligns with previous studies conducted on young adults globally and in Turkey, highlighting an increasing prevalence of obesity and high energy intake which establishes an environment for nutrition [41, 42]. Unless interventions are implemented, these individuals carry these risk factors into future decades, increasing their susceptibility to widespread NCDs such as cardiovascular diseases, diabetes, and cancer. Therefore, targeted interventions are necessary to encourage healthier eating and physical activity patterns, especially during Ramadan.

The study has several strengths. This is the first nationwide study performed to assess the dietary changes associated with Ramadan fasting among adult Turkish Muslims and their influence on body weight alterations within the studied community and our data were collected from residents in more than 10 cities in the country. Moreover, our study adheres to the recommendation by Abdelrahim et al. [9], including a diverse population of 775 males and 894 females, highlighting the importance of examining the impact of observing RIF on both sexes. However, it is important to acknowledge that this study has several limitations. Firstly, the data collected relies on self-reporting, which can introduce biases and inaccuracies in the responses. Furthermore, factors such as sleep quality and stress levels, which could influence dietary habits and weight changes, were not included in our analysis. Moreover, the study focused on a specific population during a unique period of quarantine and COVID-19, which may have influenced dietary traditions during Ramadan, and may have altered how people planned their meals, with potential implications for overeating during nonfasting hours, which could have contributed to the observed weight changes in our study [38, 39]. Therefore, the findings may not apply to other populations or regions, and caution should be exercised when generalizing the results. To address these limitations, future research should aim to include a more comprehensive range of factors that influence weight changes during Ramadan, such as sleep patterns, stress levels, and mental health. Longitudinal studies could provide insight into the long-term effects of Ramadan fasting on body weight and dietary habits across different populations. Additionally, qualitative studies exploring individuals' experiences and challenges during Ramadan could yield valuable information to inform targeted interventions for healthier eating behaviors.

In conclusion, this study highlights the critical need for mindful eating and balanced nutrition during Ramadan. Public health interventions that focus on educating individuals about healthy dietary practices during this holy month could play a vital role in combating the rising prevalence of obesity. By promoting awareness of the importance of nutritious food choices and physical activity, these initiatives can empower individuals to utilize Ramadan not only as a period of spiritual reflection but also as an opportunity for weight management and overall health improvement.

## Figures and Tables

**Table 1:** Background characteristics of study participants.

Main variable	Variable categories	Number of fasting days observed			*p* value	Total
		1–10	11–20	21–30		
Sex	Female	13	237	644	< 0.001	894
	Male	11	19	745		775

Age group (years)	18–24	13	173	901	0.02	1087
	25–34	6	62	381		449
	35–44	4	21	75		100
	45–54	0	0	22		22
	55–64	1	0	8		9
	65–74	0	0	2		2

Marital status	Divorced	1	3	9	0.008	13
	Married	7	47	289		343
	Single	16	201	1087		1304
	Widowed	0	5	4		9

Area of living	City	23	233	1287	0.624	1543
	Town	1	12	64		77
	Village	0	11	38		49

With whom living during Ramadan	Alone	3	12	105	< 0.001	120
	With a friend	0	12	182		194
	With family	21	232	1102		1355

Perceived weight status	Extreme overweight or obese	4	9	34	0.004	47
	Normal weight	12	126	698		836

**Table 2:** Weight change and associated factors during factors.

Main variable	Variable categories	No weight change		Weight loss		Weight gain		*p* value
		618 (37%)		737 (44.2%)		314 (18.8%)		
		*n*	% (Of variable category)	*n*	% (Of variable category)	*n*	% (Of variable category)	
Sex	Female	311	34.8	410	45.9	173	19.4	0.124
	Male	307	39.6	327	42.2	141	18.2	

Age group (year)	18–24	420	38.6	444	40.8	223	20.5	0.005
	25–34	158	35.2	214	47.7	77	17.1	
	35–44	28	28.0	60	60.0	12	12.0	
	45–54	10	45.5	11	50.0	1	4.5	
	55–64	2	11.8	6	35.2	9	52.9	
	65–75	0	0.00	2	100.0	0	0.0	

Physical activity	Not changed	289	43.4	262	39.3	115	17.3	< 0.001
	Decreased	258	32.8	362	46.1	166	21.1	
	Increased	71	32.9	113	52.3	32	14.8	

Snacking between *Iftar* and *Suhoor*	Infrequent	345	35.5	473	48.6	155	15.9	< 0.001
	Frequent	273	39.3	264	38.0	158	22.7	

Water consumption	Insufficient	146	33.9	202	46.9	83	19.3	0.272
	Sufficient	472	38.2	535	43.2	230	18.6	

**Table 3:** Binary logistic regression showing factors associated with weight gain during Ramadan.

Main variable	Variable categories	*p* value	AOR	95% confidence interval for exposure (B)	
Sex	Male	Ref			
	Female	0.312	1.155	0.8734	1.528

Age	18–24 years	Ref			
	35–34 years	0.792	0.958	0.6934	1.322
	35–44 years	0.561	0.813	0.4038	1.636
	45–54 years	0.106	0.181	0.0228	1.437
	55–64 years	0.991	1.015	0.0902	11.421
	65–74 years	< 0.001	0.142	0.1421	0.142

Physical activity	Not changed	Ref			
	Decreased	0.001	1.618	1.2064	2.17
	Increased	0.525	1.166	0.7264	1.872

Water consumption	Insufficient intake	Ref			
	Sufficient intake	0.281	0.84	0.6109	1.154

Abbreviation: AOR, adjusted odds ratio.

**Table 4:** Binary logistic regression showing adjusted ORs for factors associated with weight gain during Ramadan.

Dietary variable	*p* value	AOR	95% confidence interval for exposure (B)	
Increased red meat consumption	0.308	1.188	0.853	1.653
Increased salty food consumption	0.15	1.463	0.872	2.456
Increased sugary beverage consumption	0.003	1.738	1.208	2.502
Increased fried food consumption	0.435	1.185	0.775	1.811
Increased fat consumption	0.004	1.878	1.224	2.883
The reference category is no change (−0.5–+0.5)				

Abbreviation: AOR, adjusted odds ratio.

## Data Availability

The data that support the findings of this study are available on request from the corresponding author. The data are not publicly available due to privacy or ethical restrictions.
