# Correction to “Body Weight Changes During Ramadan Intermittent Fasting: A Cross‐Sectional Study of Healthy Adults in Turkey”

**DOI:** 10.1155/jnme/9850164

**Published:** 2026-08-27

A. Mektebi, M. A. Bozlar, N. Kanjo, et al., “Body Weight Changes During Ramadan Intermittent Fasting: A Cross‐Sectional Study of Healthy Adults in Turkey,” *Journal of Nutrition and Metabolism*, 2025 (2025), 8851660, https://doi.org/10.1155/jnme/8851660.

In the above‐mentioned article, there was an error in affiliation number 6. Specifically “*Applied Sciences Private University*” should have read “*Applied Science Private University*”. The corrected affiliation appears below:

Department of Clinical Nutrition and Dietetics, Faculty of Allied Medical Sciences, Applied Science Private University, Amman, Jordan.

This has been corrected in the online version accordingly.

We apologize for this error.

